# Synchronization in Finite-Time of Delayed Fractional-Order Fully Complex-Valued Dynamical Networks via Non-Separation Method

**DOI:** 10.3390/e24101460

**Published:** 2022-10-13

**Authors:** Qiaokun Kang, Qingxi Yang, Jing Yang, Qintao Gan, Ruihong Li

**Affiliations:** Shijiazhuang Campus, Army Engineering University, Shijiazhuang 050003, China

**Keywords:** finite-time synchronization, fractional-order complex networks, fully complex-valued dynamical networks, delay

## Abstract

The finite-time synchronization (FNTS) problem for a class of delayed fractional-order fully complex-valued dynamic networks (FFCDNs) with internal delay and non-delayed and delayed couplings is studied by directly constructing Lyapunov functions instead of decomposing the original complex-valued networks into two real-valued networks. Firstly, a mixed delay fractional-order mathematical model is established for the first time as fully complex-valued, where the outer coupling matrices of the model are not restricted to be identical, symmetric, or irreducible. Secondly, to overcome the limitation of the use range of a single controller, two delay-dependent controllers are designed based on the complex-valued quadratic norm and the norm composed of its real and imaginary parts’ absolute values, respectively, to improve the synchronization control efficiency. Besides, the relationships between the fractional order of the system, the fractional-order power law, and the settling time (ST) are analyzed. Finally, the feasibility and effectiveness of the control method designed in this paper are verified by numerical simulation.

## 1. Introduction

In recent years, as a characteristic collective behavior of complex networks, the synchronization problem of complex networks has received more and more attention from different fields because of its outstanding potential applications and outstanding achievements in nature, social, and technological fields [1,2,3,4]. The complexity of the system can be expressed by entropy. The more complex the system, the higher the entropy is. In the process of controlling the system to achieve synchronization, the entropy value will also decrease. In practice, it is usually hoped to realize faster synchronization or even finite-time synchronization for complex networks [5]. Many academics and researchers have been interested in the FNTS of complex networks, and there have been numerous good achievements in this area [6,7,8,9,10,11,12,13,14].

Compared with integer-order differential equations, fractional calculus is more suitable for describing the memory and genetic characteristics of various materials and dynamic processes [15,16,17,18]. The advantage of the Caputo fractional derivative is that the initial conditions of fractional differential equations with Caputo derivatives are similar to those of integral differential equations. Therefore, introducing fractional order into complex networks has theoretical and practical significance. The research on FNTS problems of fractional-order complex networks (FCNs) has become the focus and research hotspot of engineers, technicians, and scientists. For example, in [19], the FNTS for a class of FCNs was investigated by using the hybrid feedback control technique. In [20], the FNTS of FCNs with a strongly connected topology was studied. The FNTS problem between different dimensional fractional-order complex dynamical networks was investigated in [21]. In [22], the FNTS of FCNs was studied via intermittent control. In the above literature about the FNTS of FCNs, the models were all assumed to be real-valued (RV) models.

However, compared with RV dynamic networks, because dynamic systems in complex spaces can develop in different directions, CV dynamic networks allow the transmitted signals to obtain more comprehensive versatility and anti-attack performance [23]. Therefore, it is fascinating to introduce complex values into complex dynamical networks because of their practical applications in engineering technology fields [24]. By decomposing the CV systems into two RV subsystems, the synchronization or stability problems for fractional-order CV complex networks were extensively investigated in [25,26,27,28,29,30,31,32], respectively. Although the separation technique is practical, the dimension of two RV systems is double that of the originals, significantly increasing the complexity and triviality of theoretical analysis and mathematical derivation. As a result, the accuracy and simplicity of the theoretical results are low by the separation technique. Furthermore, due to the high difficulty of the model, it is not easy to transform the CV system into two RV systems in a realistic operational procedure. Hence, in [33], unlike the traditional separation method, the FNTS problem for fractional-order CV dynamical networks was investigated by introducing signum functions for complex numbers and complex-valued vectors. Similarly, in [34], Xu et al. discussed FNTS for fractional-order complex-valued coupled systems based on the complex variable function instead of the separation approach. However, the coupling strengths and inner and outer coupling matrices of the mathematical model considered in [33,34] were RV, although the state variables and system function were CV. Integrating fully CV coupling strengths and couplings into synchronization studies for complex networks is more realistic and requires more extensive analysis. To overcome this bottleneck, Zheng et al. [35] designed the power law control strategies for CV networks by introducing the signum function in the complex domain and studied the FNTS for a class of fully CV neural networks In [36], based on the introduced CV vector signum function, the complex value control strategy was directly designed for the fully CV integer-order complex networks. The FNTS and fixed-time synchronization problems of the integer-order fully CV network were studied.

On the other hand, it is well known that delay is a common phenomenon in the real world [37,38,39]. Time delay widely exists in complex network systems such as medicine circulation systems, population dynamics models, disease infection models, neural network models, communication networks, power networks, economic systems, etc. In complex networks, there exist internal delay and coupling delay, which will show finite speed and propagation, as well as the impact of traffic congestion on node behavior, respectively [40]. The existence of time delay will increase the difficulty of analysis. When the controlled object has internal delay in the control system, the control difficulty of the system will increase. Besides, time delay systems have a richer dynamic behavior, which is more widely used for secure communication. Therefore, it is meaningful to study dynamic networks with internal delay and coupling delay. However, the FNTS results of the fully CV dynamical networks mentioned above do not demonstrate internal delay and coupling delay. Introducing internal delay and coupling delay into complex dynamics networks needs further analysis. Regretfully, as far as we know, there are few or no results on FNTS of FFCDNs with internal delay, as well as linearly non-delayed and delayed couplings. Our current research is motivated by this condition. To better illustrate the contribution of our study, we compared our paper with other similar papers published in the last three years. The differences are shown in Table 1, where fixed-time synchronization and adaptive synchronization are abbreviated as FXTS and ADS, respectively. The signum ✔ means the object is included in the paper. The signum ✕ means the object is not included in the paper.

Through comparison, it can be found that it is difficult and challenging to comprehensively study a class of fully fractional-order CV dynamic networks with internal delay, no delay, and time delay coupling. The main contribution of this research may be described as follows:

(1) The complex dynamical networks studied in this paper are novel. The state variables, system function, coupling strengths, inner coupling matrices, and outer coupling matrices in the considered dynamical networks are all CV. In addition, the mathematical model is the fractional-order case, which is more consistent with practical applications.

(2) Lyapunov functions are constructed based on the quadratic norm and the new norm composed of the absolute value norm by introducing the signum function. The fractional-order complex-valued networks do not need to be separated into real and imaginary parts, which reduces conservatism, complexity, and trivialness.

(3) In order to overcome the limitation of a single controller, two kinds of different controllers (based on the quadratic norm and 1-norm for a complex vector, respectively) are deployed in realizing FNTS, and a series of straightforward and flexible synchronization criteria is acquired.

(4) Compared with the separation method, which needs to apply controllers to the multiple separate systems, the control strategy in this paper is simpler and more efficient, can effectively reduce the cost, and has high practical application value.

The following is the structure of the paper. In Section 2, the preliminaries and the model description are given; in Section 3, two different controllers are proposed to ensure FNTS for the addressed delayed FFCDNs; numerical simulations are presented in Section 4 to illustrate the validity and practicality of the proposed theoretical solutions; the conclusion is given in Section 5.

Notations: Throughout this study, R and C represent the real field and complex field, respectively. $R^{+}$ denotes the positive real field. $C^{n}$ symbolizes the *n*-dimensional complex space. For any v=p+iq∈C, $\bar{v}=p-iq$ denotes the conjugate of *v*, ${\left|v\right|}_{1}=\left|p\right|+\left|q\right|$, ${\left|v\right|}_{2}=\sqrt{\bar{v}v}$, where i meets $i=\sqrt{-1}$, and p,q∈R are the real and imaginary parts of *v*, respectively, that is Re(v)=p and Im(v)=q. [v]=sign(Re(v))+isign(Im(v)) is said to be the signum function of *v*. For any $v=\left(v_{1},v_{2},\cdots ,v_{n}\right)\in C^{n}$, v=Re(v)+iIm(v), $v^{H}$ denotes its conjugate transposition, ∥v∥1=∥Re(v)∥1+i∥Im(v)∥1, ${∥v∥}_{2}=\sqrt{v^{H}v}$, and [v]=sign(Re(v1))+isign(Im(v1)),⋯,sign(Re(vn))+isign(Im(vn)T. $C^{m\times n}$ denotes the set of all m×n-dimensional complex matrices. $I_{N}$ denotes the *n*-dimensional column vector with each element equal to 1, and $E_{N}$ represents the *n*-dimensional diagonal identity matrix. The notation $C^{n}\left(\left[t_{0},+\infty \right),C\right)$ denotes the family of all continuous *n*-differential functions from $\left[t_{0},+\infty \right)$ into C.

## 2. Preliminaries and Model Description

In this work, we selected the α-order Caputo derivative to depict the dynamic behavior of delayed FFCDNs.

**Definition** **1**  ([47]). *For an integrable function $f\left(t\right):\left[t_{0},+\infty \right)\to C$, its α-order fractional integral is defined as*

$${}_{t_{0}}I_{t}^{\alpha }=\frac{1}{\Gamma (\alpha )}\int_{t_{0}}^{t}{\left(t-s\right)}^{\alpha -1}f\left(s\right)ds,\;t\geq t_{0},$$

*where Γ(·) is the Gamma function and α>0.*

**Definition** **2**  ([48]). *For $f\in C^{h}(\left[t_{0},+\infty \right),C$), its α-order Caputo derivative is defined as*

$${}_{t_{0}}^{C}D_{t}^{\alpha }f\left(t\right)=\frac{1}{\Gamma (h-\alpha )}\int_{t_{0}}^{t}\frac{f^{\left(h\right)}\left(s\right)}{{\left(t-s\right)}^{\alpha }}ds,\;t\geq t_{0},$$

*where h is a positive integer such that h−1<α<h. Especially, if 0<α<1, then*

$${}_{t_{0}}^{C}D_{t}^{\alpha }f\left(t\right)=\frac{1}{\Gamma (1-\alpha )}\int_{t_{0}}^{t}\frac{f^{\prime }\left(s\right)}{{\left(t-s\right)}^{\alpha }}ds.$$

**Remark** **1.**  *Because of the super singularity of the Riemann–Liouville fractional derivative, it is limited in the application of engineering and physical modeling. The weak singularity of the Caputo fractional derivative operator solves the initial value problem in the definition of Riemann–Liouville fractional calculus. It is widely used in the modeling process in practical applications. Therefore, the Caputo fractional derivative was selected in this paper.*

**Definition** **3**  ([33]). *For any real numbers m,q>0, the integral*

$$\int_{0}^{1}x^{m-1}{\left(1-x\right)}^{q-1}dx,$$

*is called the Beta function, denoted by $\tilde{\Gamma }\left(m,q\right)$. It is easy to see that*

$$\tilde{\Gamma }\left(m,q\right)=\frac{\Gamma (m)\Gamma (q)}{\Gamma (m+q)}.$$

The delayed FFCDNs can be described as follows:

(1) $$\begin{array}{cc} {}_{t_{0}}^{C}D_{t}^{\alpha }x_{i}\left(t\right)= & f\left(x_{i}\left(t\right),x_{i}\left(t-\tau_{1}\right)\right)+c_{1}\sum_{j=1}^{N}a_{ij}G_{1}x_{j}\left(t\right)+c_{2}\sum_{j=1}^{N}b_{ij}G_{2}x_{j}\left(t-\tau_{2}\right),\;i=1,2,\cdots ,N, \end{array}$$

where 0<α<1, α∈(0,1), $x_{i}\left(t\right)={\left(x_{i1}\left(t\right),x_{i2}\left(t\right),\cdots ,x_{in}\left(t\right)\right)}^{T}\in C^{n}$ represents the state vector of the *i*th node at time *t*, $f:C^{n}\times C^{n}\to C^{n}$ is a nonlinear vector function, $c_{l}\in C$(l=1,2) is the coupling strength, $G_{l}=\mathrm{diag}\left(\delta_{1}^{\left(l\right)},\delta_{2}^{\left(l\right)},\cdots ,\delta_{n}^{\left(l\right)}\right)\in C^{n\times n}$(l=1,2) is the inner matrix linking the coupled variables, $\tau_{1}$ is the internal delay occurring inside the dynamical node, $\tau_{2}$ represents the coupling delay, and $A={\left(a_{ij}\right)}_{N\times N}$ and $B={\left(b_{ij}\right)}_{N\times N}$ represent the topological structure of FFCDNs without and with time delays, respectively. The initial conditions associated with Equation (1) are given as $x_{i}\left(s\right)=\varphi_{i}\left(s\right)\in C\left(\left[-\tau ,0\right],C^{n}\right),i=1,2,3\ldots N,$ where $C(\left[-\tau ,0\right],C^{n})$ represents the set of all n-dimensional continuous differentiable functions defined on the interval [−τ,0] and τ = max{$\tau_{1},\tau_{2}$}.

The topological structure of delayed FFCDNs should match the following criteria: if node *i* and node *j*(i≠j) have a link, then $a_{ij},b_{ij}\neq 0\in C$; else $a_{ij}=b_{ij}=0$(i≠j), and the diagonal elements are

$$a_{ii}=-\sum_{j=1,j\neq i}^{N}a_{ij},\;b_{ii}=-\sum_{j=1,j\neq i}^{N}b_{ij},\;i=1,2,\cdots ,N.$$

**Definition** **4.**  *The set $S=\{{\left(x_{1}^{T}\left(t\right),x_{2}^{T}\left(t\right),\cdots ,x_{N}^{T}\left(t\right)\right)}^{T}\in C^{n\times N}:x_{i}\left(t\right)=x_{j}\left(t\right)=s\left(t\right)\in C^{n}$ for all i,j=1,2,⋯,N} is known as the synchronization manifold of system (1), and s(t) is known as the synchronous state of (1).*

It follows from (1) and the definitions of *A* and *B* that

(2) $${}_{t_{0}}^{C}D_{t}^{\alpha }s\left(t\right)=f\left(s\left(t\right),s\left(t-\tau_{1}\right)\right).$$

Define the error states as $e_{i}\left(t\right)=x_{i}\left(t\right)-s\left(t\right)$ with i=1,2,⋯,N. It is clear that

(3) $$\begin{array}{cc} {}_{t_{0}}^{C}D_{t}^{\alpha }e_{i}\left(t\right)=\; & g\left(e_{i}\left(t\right),e_{i}\left(t-\tau_{1}\right)\right)+c_{1}\sum_{j=1}^{N}a_{ij}G_{1}e_{j}\left(t\right)+c_{2}\sum_{j=1}^{N}b_{ij}G_{2}e_{j}\left(t-\tau_{2}\right)+u_{i}\left(t\right), \end{array}$$

where i=1,2,⋯,N, $g\left(e_{i}\left(t\right),e_{i}\left(t-\tau_{1}\right)\right)=f\left(x_{i}\left(t\right),x_{i}\left(t-\tau_{1}\right)\right)-f\left(s\left(t\right),s\left(t-\tau_{1}\right)\right)$, and $u_{i}\left(t\right)$ is a controller that will be created later.

The following assumptions, definitions, and lemmas are required to reach our major conclusions.

**Assumption** **A1.**  *For the vector-valued function $f:C^{n}\times C^{n}\to C^{n}$, there exist $\eta_{p}$, $\zeta_{p}$ > 0 (p=1,2), such that*

$$\begin{array}{cc} & ∥f\left(x\left(t\right),x\left(t-\tau_{1}\right)\right)-f\left(s\left(t\right),s\left(t-\tau_{1}\right)\right){∥}_{p}\\ \leq & \eta_{p}{∥x\left(t\right)-s\left(t\right)∥}_{p}+\zeta_{p}{∥x\left(t-\tau_{1}\right)-s\left(t-\tau_{1}\right)∥}_{p} \end{array}$$

*for any $x\left(t\right),s\left(t\right)\in C^{n}$ and t≥0, where ${∥\cdot ∥}_{1}$ denotes the 1-norm and ${∥\cdot ∥}_{2}$ denotes the Euclidean norm.*

**Remark** **2.**  *Assumption 1 is a reasonable requirement for the synchronization of complex networks. This assumption is not very strict, but relatively broad. Many classical chaotic systems with or without time delay satisfy this assumption, such as the Lorenz system, Chen system, and Li system [49].*

**Definition** **5.**  *System (1) is considered to achieve FNTS if there exists the ST $T(e\left(t_{0}\right))$, which is dependent on the initial synchronization error, such that $\lim_{t\to T(e\left(t_{0}\right))}{∥e_{i}\left(t\right)∥}_{p}=0$ and $e_{i}\left(t\right)\equiv 0$ for $t>T(e\left(t_{0}\right))$, i=1,2,⋯,N, where p=1,2, $e\left(t_{0}\right)={\left(e_{1}^{T}\left(t_{0}\right),e_{2}^{T}\left(t_{0}\right),\cdots ,e_{N}^{T}\left(t_{0}\right)\right)}^{T}$.*

**Lemma** **1**  ([50]). *If $\lambda \left(t\right)\in C^{n}$ is differentiable, for $t\geq t_{0}$ and 0<α<1, the following inequality holds:*

$${}_{t_{0}}^{C}D_{t}^{\alpha }\lambda^{H}\left(t\right)\lambda \left(t\right)\leq \lambda^{H}{\left(t\right)}_{t_{0}}^{C}D_{t}^{\alpha }\lambda \left(t\right)+\left({}_{t_{0}}^{C}D_{t}^{\alpha }\lambda^{H}\left(t\right)\right)\lambda \left(t\right).$$

**Lemma** **2**  ([36]). *For any u∈C, μ(t), $\Lambda \left(t\right)\in C^{n}$, the following statements are true for α∈(0,1).*

(1)
u+u¯=2Re(u)≤|u|2≤|u|1.

(2)
$\mu^{H}\left(t\right)\left[\Lambda \left(t\right)\right]+{\left[\Lambda \left(t\right)\right]}^{H}\mu \left(t\right)\leq 2{∥\mu \left(t\right)∥}_{1}.$

(3)
$\Lambda^{H}\left(t\right)\left[\Lambda \left(t\right)\right]+{\left[\Lambda \left(t\right)\right]}^{H}\Lambda \left(t\right)={2∥\Lambda \left(t\right)∥}_{1}\geq 2{∥\Lambda \left(t\right)∥}_{2}.$

(4)
${}_{t_{0}}^{C}D_{t}^{\alpha }\left(\Lambda^{H}\left(t\right)\left[\Lambda \left(t\right)\right]+{\left[\Lambda \left(t\right)\right]}^{H}\Lambda \left(t\right)\right)\leq {\left[\Lambda \left(t\right)\right]}^{H}{}_{t_{0}}^{C}D_{t}^{\alpha }\Lambda \left(t\right)+\left({}_{t_{0}}^{C}D_{t}^{\alpha }\Lambda^{H}\left(t\right)\right)\left[\Lambda \left(t\right)\right].$

**Lemma** **3**  ([36]). *For any μ, Λ∈C, the following inequality is true:*

uΛ[Λ]¯+uΛ¯[Λ]≤2Re(μ)+|Im(μ)||Λ|1.

**Lemma** **4**  ([51]). *Assume that $\lambda_{i}\geq 0$ for i=1,2,⋯,n, p>1, 0<q<1, then the following inequalities hold:*

$$\sum_{i=1}^{n}\lambda_{i}^{q}\geq {\left(\sum_{i=1}^{n}\lambda_{i}\right)}^{q},\;\sum_{i=1}^{n}\lambda_{i}^{p}\geq n^{1-p}{\left(\sum_{i=1}^{n}\lambda_{i}\right)}^{p}.$$

**Lemma** **5**  ([33]). *Suppose that there exist positive constants γ∈(0,α) and λ supposing that*

(4) $${}_{t_{0}}^{C}D_{t}^{\alpha }V\left(t\right)\leq -\lambda V^{\gamma }\left(t\right),\;V\left(t\right)\in R^{+},$$

then $\lim_{t\to T^{*}}V\left(t\right)=0$ and V(t)≡0 for all $t\geq T^{*}$, in which

(5) $$T^{*}=t_{0}+{\left(\frac{\alpha }{\lambda }V^{\alpha -\gamma }\left(t_{0}\right)B\left(\alpha ,1-\gamma \right)\right)}^{1/\alpha }.$$

## 3. Main Results

The FNTS problems for a class of FFCDNs with internal delay and linearly non-delayed and delayed couplings were explored by designing two different controllers. The following are the key results.

**Theorem** **1.**  *Based on Assumption 1, the control law is constructed as follows:*

(6) $$\begin{array}{cc} u_{i}\left(t\right)= & -d_{i}e_{i}\left(t\right)-\beta \left[e_{i}\left(t\right)\right]{∥e_{i}\left(t\right)∥}_{2}^{\gamma }-\frac{1}{2}\sum_{r=1}^{2}\varepsilon_{i}^{\left(r\right)}e_{i}\left(t-\tau_{r}\right), \end{array}$$

*where $d_{i},\beta ,\varepsilon_{i}^{\left(1\right)},\varepsilon_{i}^{\left(2\right)}>0$, 0<γ<2α−1, i=1,2,⋯,N, satisfying*

(7) $$\left\{\begin{array}{c} 2\eta_{2}E_{N}+c_{1}\delta_{k}^{\left(1\right)}A+\bar{c_{1}}\bar{\delta_{k}^{\left(1\right)}}A^{H}-2D\leq 0,\\ \zeta_{2}E_{N}-\Pi^{\left(1\right)}\leq 0,\\ c_{2}\delta_{k}^{\left(2\right)}B-\Pi^{\left(2\right)}\leq 0,\\ \bar{c_{2}}\bar{\delta_{k}^{\left(2\right)}}B^{H}-\Pi^{\left(2\right)}\leq 0, \end{array}\right.$$

*where $D=\mathrm{diag}(d_{1},d_{2},\cdots ,d_{N})$, $\Pi^{\left(r\right)}=\mathrm{diag}\left(\varepsilon_{1}^{\left(r\right)},\varepsilon_{2}^{\left(r\right)},\cdots ,\varepsilon_{N}^{\left(r\right)}\right)$, (r=1,2), for all k=1,2,⋯,n. Then, the controlled FFCDNs (3) is said to achieve FNTS, and the ST is estimated as*

(8) $$T\leq T_{1}=t_{0}+{\left(\frac{\alpha ∥e\left(t_{0}\right){∥}_{2}^{2(\alpha -\tilde{\gamma })}}{2^{\alpha }\beta }\tilde{\Gamma }\left(\alpha ,1-\tilde{\gamma }\right)\right)}^{1/\alpha },$$

*where $\tilde{\gamma }=\left(1+\gamma \right)/2$.*

**Proof.**  Define the Lyapunov function:

(9) $$V_{1}\left(t\right)=\frac{1}{2}\sum_{i=1}^{N}e_{i}^{H}\left(t\right)e_{i}\left(t\right).$$

Computing the fractional-order derivative of $V_{1}\left(t\right)$ along the solutions of (3), it follows from Lemma 1 that

(10) $$\begin{array}{cc} {}_{t_{0}}^{C}D_{t}^{\alpha }V_{1}\left(t\right)\leq & \frac{1}{2}\sum_{i=1}^{N}\left(e_{i}^{H}\left(t\right)g\left(e_{i}\left(t\right),e_{i}\left(t-\tau_{1}\right)\right)+g^{H}\left(e_{i}\left(t\right),e_{i}\left(t-\tau_{1}\right)\right)e_{i}\left(t\right)\right)\\ & +\frac{1}{2}\sum_{i=1}^{N}\sum_{j=1}^{N}\left(e_{i}^{H}\left(t\right)c_{1}a_{ij}G_{1}e_{j}\left(t\right)+e_{j}^{H}\left(t\right)\bar{c_{1}a_{ij}}G_{1}^{H}e_{i}\left(t\right)\right)\\ & +\frac{1}{2}\sum_{i=1}^{N}\sum_{j=1}^{N}\left(e_{i}^{H}\left(t\right)c_{2}b_{ij}G_{2}e_{j}\left(t-\tau_{2}\right)+e_{j}^{H}\left(t-\tau_{2}\right)\bar{c_{2}b_{ij}}G_{2}^{H}e_{i}\left(t\right)\right)\\ & -\frac{1}{2}\sum_{r=1}^{2}\sum_{i=1}^{N}\varepsilon_{i}^{\left(r\right)}\left(e_{i}^{H}\left(t\right)e_{i}\left(t-\tau_{r}\right)+e_{i}^{H}\left(t-\tau_{r}\right)e_{i}\left(t\right)\right)\\ & -\frac{\beta }{2}\sum_{i=1}^{N}\left({\left[e_{i}\left(t\right)\right]}^{H}e_{i}\left(t\right)+e_{i}^{H}\left(t\right)\left[e_{i}\left(t\right)\right]\right){∥e_{i}\left(t\right)∥}_{2}^{\gamma }-\sum_{i=1}^{N}d_{i}e_{i}^{H}\left(t\right)e_{i}\left(t\right). \end{array}$$

On the basis of Assumption 1 and Lemma 2, we have

(11) $$\begin{array}{cc} & \frac{1}{2}\sum_{i=1}^{N}\left(e_{i}^{H}\left(t\right)g\left(e_{i}\left(t\right),e_{i}\left(t-\tau_{1}\right)\right)+g^{H}\left(e_{i}\left(t\right),e_{i}\left(t-\tau_{1}\right)\right)e_{i}\left(t\right)\right)\\ = & \frac{1}{4}\sum_{i=1}^{N}(e_{i}^{H}\left(t\right)g\left(e_{i}\left(t\right),e_{i}\left(t-\tau_{1}\right)\right)+g^{H}\left(e_{i}\left(t\right),e_{i}\left(t-\tau_{1}\right)\right)e_{i}\left(t\right)\\ & +\sum_{i=1}^{N}\left(g^{H}\left(e_{i}\left(t\right),e_{i}\left(t-\tau_{1}\right)\right)e_{i}\left(t\right)+e_{i}^{H}\left(t\right)g\left(e_{i}\left(t\right),e_{i}\left(t-\tau_{1}\right)\right)\right)\\ \leq & \frac{1}{2}\sum_{i=1}^{N}\left(∥e_{i}^{H}{\left(t\right)∥}_{2}∥g\left(e_{i}\left(t\right),e_{i}\left(t-\tau_{1}\right)\right){∥}_{2}+∥g^{H}\left(e_{i}\left(t\right),e_{i}\left(t-\tau_{1}\right)\right){∥}_{2}{∥e_{i}\left(t\right)∥}_{2}\right)\\ \leq & \eta_{2}\sum_{i=1}^{N}\sum_{k=1}^{n}\bar{e_{ik}\left(t\right)}e_{ik}\left(t\right)+\frac{\zeta_{2}}{2}\sum_{i=1}^{N}\sum_{k=1}^{n}\left(\bar{e_{ik}\left(t\right)}e_{ik}\left(t-\tau_{1}\right)+\bar{e_{ik}\left(t-\tau_{1}\right)}e_{ik}\left(t\right)\right)\\ = & \eta_{2}\sum_{k=1}^{n}e_{k}^{H}\left(t\right)e_{k}\left(t\right)+\frac{\zeta_{2}}{2}\sum_{k=1}^{n}\left(e_{k}^{H}\left(t\right)e_{k}\left(t-\tau_{1}\right)+e_{k}^{H}\left(t-\tau_{1}\right)e_{k}\left(t\right)\right). \end{array}$$

From Lemma 2, we also have

(12) $$\begin{array}{cc} & \frac{1}{2}\sum_{i=1}^{N}\sum_{j=1}^{N}\left(e_{i}^{H}\left(t\right)c_{1}a_{ij}G_{1}e_{j}\left(t\right)+e_{j}^{H}\left(t\right)\bar{c_{1}a_{ij}}G_{1}^{H}e_{i}\left(t\right)\right)\\ = & \frac{1}{2}\sum_{i=1}^{N}\sum_{j=1}^{N}\sum_{k=1}^{n}\left(\bar{e_{ik}\left(t\right)}c_{1}a_{ij}\delta_{k}^{\left(1\right)}e_{jk}\left(t\right)+\bar{e_{jk}\left(t\right)}\bar{c_{1}a_{ij}\delta_{k}^{\left(1\right)}}e_{ik}\left(t\right)\right)\\ = & \frac{1}{2}\sum_{k=1}^{n}e_{k}^{H}\left(t\right)\left(c_{1}\delta_{k}^{\left(1\right)}A+\bar{c_{1}}\bar{\delta_{k}^{\left(1\right)}}A^{H}\right)e_{k}\left(t\right), \end{array}$$

and

(13) $$\begin{array}{cc} & \frac{1}{2}\sum_{i=1}^{N}\sum_{j=1}^{N}\left(e_{i}^{H}\left(t\right)c_{2}b_{ij}G_{2}e_{j}\left(t-\tau_{2}\right)+e_{j}^{H}\left(t-\tau_{2}\right)\bar{c_{2}b_{ij}}G_{2}^{H}e_{i}\left(t\right)\right)\\ = & \frac{1}{2}\sum_{i=1}^{N}\sum_{j=1}^{N}\sum_{k=1}^{n}\left(\bar{e_{ik}\left(t\right)}c_{2}b_{ij}\delta_{k}^{\left(2\right)}e_{jk}\left(t-\tau_{2}\right)+\bar{e_{jk}\left(t-\tau_{2}\right)}\bar{c_{2}b_{ij}\delta_{k}^{\left(2\right)}}e_{ik}\left(t\right)\right)\\ = & \frac{1}{2}\sum_{k=1}^{n}\left(e_{k}^{H}\left(t\right)\left(c_{2}\delta_{k}^{\left(2\right)}B\right)e_{k}\left(t-\tau_{2}\right)+e_{k}^{H}\left(t-\tau_{2}\right)\left(\bar{c_{2}}\bar{\delta_{k}^{\left(2\right)}}B^{H}\right)e_{k}\left(t\right)\right). \end{array}$$

Furthermore, according to Lemmas 2 and 4, we can obtain:

(14) $$\begin{array}{cc} & -\frac{\beta }{2}\sum_{i=1}^{N}\left({\left[e_{i}\left(t\right)\right]}^{H}e_{i}\left(t\right)+e_{i}^{H}\left(t\right)\left[e_{i}\left(t\right)\right]\right){∥e_{i}\left(t\right)∥}_{2}^{\gamma }\leq -\beta {\left(\sum_{i=1}^{N}{∥e_{i}\left(t\right)∥}_{2}^{2}\right)}^{(1+\gamma )/2}. \end{array}$$

Substituting (11)–(14) into (10), we can obtain

(15) $$\begin{array}{cc} {}_{t_{0}}^{C}D_{t}^{\alpha }V_{1}\left(t\right)\leq & \frac{1}{2}\sum_{k=1}^{n}e_{k}^{H}\left(t\right)\left(2\eta_{2}E_{N}+c_{1}\delta_{k}^{\left(1\right)}A+\bar{c_{1}}\bar{\delta_{k}^{\left(1\right)}}A^{H}-2D\right)e_{k}\left(t\right)-\beta {\left(\sum_{i=1}^{N}{∥e_{i}\left(t\right)∥}_{2}^{2}\right)}^{(1+\gamma )/2}\\ & +\frac{1}{2}\sum_{k=1}^{n}\left(e_{k}^{H}\left(t\right)\left(\zeta_{2}E_{N}-\Pi^{\left(1\right)}\right)e_{k}\left(t-\tau_{1}\right)+e_{k}^{H}\left(t-\tau_{1}\right)\left(\zeta_{2}E_{N}-\Pi^{\left(1\right)}\right)e_{k}\left(t\right)\right)\\ & +\frac{1}{2}\sum_{k=1}^{n}\left(e_{k}^{H}\left(t\right)\left(c_{2}\delta_{k}^{\left(2\right)}B-\Pi^{\left(2\right)}\right)e_{k}\left(t-\tau_{2}\right)+e_{k}^{H}\left(t-\tau_{2}\right)\left(\bar{c_{2}}\bar{\delta_{k}^{\left(2\right)}}B^{H}-\Pi^{\left(2\right)}\right)e_{k}\left(t\right)\right)\\ \leq & -\beta {\left(\sum_{i=1}^{N}{∥e_{i}\left(t\right)∥}_{2}^{2}\right)}^{(1+\gamma )/2}. \end{array}$$

Based on Lemma 5, the FFCDNs (3) under the controller (6) could achieve synchronization within time $T_{1}$. The proof is accomplished. □

**Remark** **3.**  *Each part of the controller (6) has a unique contribution for FNTS of delayed FFCDNs. The delayed nonlinear dynamics and coupled configuration are compensated by the terms $-d_{i}e_{i}\left(t\right)$, $-\varepsilon_{i}^{\left(1\right)}e_{i}\left(t-\tau_{1}\right)$, and $-\varepsilon_{i}^{\left(2\right)}e_{i}\left(t-\tau_{2}\right)$; the term $-\beta \left[e_{i}\left(t\right)\right]{∥e_{i}\left(t\right)∥}_{2}^{\gamma }$ plays a key role in realizing FNTS. In addition, designing the delay-independent controllers, which are easy to implement and can achieve a better synchronization control effect, will be the authors’ future investigative direction.*

**Remark** **4.**  *In Theorem 1, the FNTS problem for a class of FFCDNs with linearly non-delayed and delayed couplings is deliberated based on the quadratic norm. On the other hand, the limitation conditions 0<γ<2α−1 and 0<α<1 are quite restrictive and may not be more realistic. Consequently, we next provide the results for FNTS in terms of a novel norm composed of the absolute values of each part.*

**Theorem** **2.**  *Based on Assumption 1, the control law is constructed as*

(16) $$\begin{array}{cc} u_{i}\left(t\right)= & -d_{i}e_{i}\left(t\right)-\beta \left[e_{i}\left(t\right)\right]{∥e_{i}\left(t\right)∥}_{1}^{\gamma }-\frac{1}{2}\sum_{r=1}^{2}\varepsilon_{i}^{\left(r\right)}\left[e_{i}\left(t\right)\right]e_{i}^{H}\left(t-\tau_{r}\right)\left[e_{i}\left(t-\tau_{r}\right)\right], \end{array}$$

*where $d_{i},\beta ,\varepsilon_{i}^{\left(1\right)},\varepsilon_{i}^{\left(2\right)}>0$, 0<γ<α, i=1,2,⋯,N, satisfying*

(17) $$\left\{\begin{array}{c} \eta_{1}E_{N}+\Xi^{\left(k\right)}-D\leq 0,\\ \zeta_{1}E_{N}-\Pi^{\left(1\right)}\leq 0,\\ \Omega^{\left(k\right)}-\Pi^{\left(2\right)}\leq 0, \end{array}\right.$$

*in which $D=\mathrm{diag}(d_{1},d_{2},\cdots ,d_{N})$, $\Pi^{\left(r\right)}=\mathrm{diag}\left(\varepsilon_{1}^{\left(r\right)},\varepsilon_{2}^{\left(r\right)},\cdots ,\varepsilon_{N}^{\left(r\right)}\right)$, (r=1,2), $\Xi^{\left(k\right)}={\left(\lambda_{ij}^{\left(k\right)}\right)}_{N\times N}$, $\Omega^{\left(k\right)}={\left(\omega_{ij}^{\left(k\right)}\right)}_{N\times N}$, and*

λij(k)=Re(c1aiiδk(1))+|Im(c1aiiδk(1))|,i=j,|c1aijδk(1)|1,i≠j,ωij(k)=|Re(c2bijδk(2))|+|Im(c2bijδk(2))|,

*for all k=1,2,⋯,n. Then, the controlled FFCDNs (3) could achieve synchronization, and the ST is estimated as*

(18) $$T\leq T_{2}=t_{0}+{\left(\frac{\alpha }{\beta }{∥e\left(t_{0}\right)∥}_{1}^{\alpha -\gamma }\tilde{\Gamma }\left(\alpha ,1-\gamma \right)\right)}^{1/\alpha }.$$

**Proof.**  Define the Lyapunov function:

(19) $$V_{2}\left(t\right)=\frac{1}{2}\sum_{i=1}^{N}\left(e_{i}^{H}\left(t\right)\left[e_{i}\left(t\right)\right]+{\left[e_{i}\left(t\right)\right]}^{H}e_{i}\left(t\right)\right)=\sum_{i=1}^{N}{∥e_{i}\left(t\right)∥}_{1}.$$

Computing the fractional-order derivative of $V_{2}\left(t\right)$ along the solutions of (3), it follows from Lemma 2 that

(20) $$\begin{array}{cc} {}_{t_{0}}^{C}D_{t}^{\alpha }V_{2}\left(t\right)\leq & \frac{1}{2}\sum_{i=1}^{N}\left({\left[e_{i}\left(t\right)\right]}^{H}g\left(e_{i}\left(t\right),e_{i}\left(t-\tau_{1}\right)\right)+g^{H}\left(e_{i}\left(t\right),e_{i}\left(t-\tau_{1}\right)\right)\left[e_{i}\left(t\right)\right]\right)\\ & +\frac{1}{2}\sum_{i=1}^{N}\sum_{j=1}^{N}\left({\left[e_{i}\left(t\right)\right]}^{H}c_{1}a_{ij}G_{1}e_{j}\left(t\right)+e_{j}^{H}\left(t\right)\bar{c_{1}a_{ij}}G_{1}^{H}\left[e_{i}\left(t\right)\right]\right)\\ & +\frac{1}{2}\sum_{i=1}^{N}\sum_{j=1}^{N}\left({\left[e_{i}\left(t\right)\right]}^{H}c_{2}b_{ij}G_{2}e_{j}\left(t-\tau_{2}\right)+e_{j}^{H}\left(t-\tau_{2}\right)\bar{c_{2}b_{ij}}G_{2}^{H}\left[e_{i}\left(t\right)\right]\right)\\ & -\frac{1}{2}\sum_{i=1}^{N}d_{i}\left({\left[e_{i}\left(t\right)\right]}^{H}e_{i}\left(t\right)+e_{i}^{H}\left(t\right)\left[e_{i}\left(t\right)\right]\right)-\beta \sum_{i=1}^{N}{\left[e_{i}\left(t\right)\right]}^{H}\left[e_{i}\left(t\right)\right]{∥e_{i}\left(t\right)∥}_{1}^{\gamma }\\ & -\frac{1}{2}\sum_{r=1}^{2}\sum_{i=1}^{N}\varepsilon_{i}^{\left(r\right)}\left({\left[e_{i}\left(t-\tau_{r}\right)\right]}^{H}e_{i}\left(t-\tau_{r}\right)+e_{i}^{H}\left(t-\tau_{r}\right)\left[e_{i}\left(t-\tau_{r}\right)\right]\right). \end{array}$$

On the basis of Assumption 1 and Lemma 2, we have

(21) 12∑i=1N[ei(t)]Hg(ei(t),ei(t−τ1))+gH(ei(t),ei(t−τ1))[ei(t)]=12∑i=1N∑k=1n[eik(t)]¯gk(ei(t),ei(t−τ1))+gk(ei(t),ei(t−τ1))¯[eik(t)]=∑i=1N∑k=1n(sign(Re(eik(t)))Regk(ei(t),ei(t−τ1))+sign(Im(eik(t)))Imgk(ei(t),ei(t−τ1)))≤∑i=1N∥g(ei(t),ei(t−τ1))∥1≤η1∑i=1N∥ei(t)∥1+ζ1∑i=1N∥ei(t−τ1)∥1=η1∑k=1nINTek(t)_+ζ1∑k=1nINTek(t−τ1)_,

where $\underline{e_{k}\left(t\right)}={\left(|e_{1k}{\left(t\right)|}_{1},|e_{2k}{\left(t\right)|}_{1},\cdots ,{\left|e_{Nk}\left(t\right)\right|}_{1}\right)}^{T}$ and $\underline{e_{k}\left(t-\tau_{1}\right)}={\left(|e_{1k}\left(t-\tau_{1}\right){|}_{1},|e_{2k}\left(t-\tau_{1}\right){|}_{1},\cdots ,{\left|e_{Nk}\left(t-\tau_{1}\right)\right|}_{1}\right)}^{T}$.

According to Lemmas 2 and 3, we also have

(22) 12∑i=1N∑j=1N[ei(t)]Hc1aijG1ej(t)+ejH(t)c1aij¯G1H[ei(t)]=12∑i=1N∑j=1N∑k=1n[eik(t)]¯c1aijδk(1)ejk(t)+ejk(t)¯c1aijδk(1)¯[eik(t)]=12∑i=1N∑k=1n[eik(t)]¯c1aiiδk(1)ejk(t)+ejk(t)¯c1aiiδk(1)¯[eik(t)]+12∑i=1N∑j=1,j≠iN∑k=1n[eik(t)]¯c1aijδk(1)ejk(t)+ejk(t)¯c1aijδk(1)¯[eik(t)]≤∑i=1N∑k=1nRe(c1aiiδk(1))+|Im(c1aiiδk(1))||ejk(t)|1+∑i=1N∑j=1,j≠iN∑k=1n|c1aijδk(1)|1|ejk(t)|1=∑i=1N∑j=1N∑k=1nλij(k)|ejk(t)|1=∑k=1nINTΞ(k)ek(t)_,

and

(23) 12∑i=1N∑j=1N[ei(t)]Hc2bijG2ej(t−τ2)+ejH(t−τ2)c2bij¯G2H[ei(t)]=12∑i=1N∑j=1N∑k=1n[eik(t)]¯c2bijδk(2)ejk(t−τ2)+ejk(t−τ2)¯c2baijδk(2)¯[eik(t)]≤∑i=1N∑j=1N∑k=1n|Re(c2bijδk(2))|+|Im(c2bijδk(2))||ejk(t−τ2)|1=∑i=1N∑j=1N∑k=1n|c2bijδk(2)||ejk(t−τ2)|1=∑i=1N∑j=1N∑k=1nωij(k)|ejk(t−τ2)|1=∑k=1nINTΩ(k)ek(t−τ2)_.

In addition, according to Lemmas 2 and 4, we can obtain

(24) $$\begin{array}{cc} & -\beta \sum_{i=1}^{N}{\left[e_{i}\left(t\right)\right]}^{H}\left[e_{i}\left(t\right)\right]∥e_{i}{\left(t\right)∥}_{1}^{\gamma }=-\beta \sum_{i=1}^{N}∥\left[e_{i}\left(t\right)\right]{∥}_{1}∥e_{i}{\left(t\right)∥}_{1}^{\gamma }\leq -\beta \sum_{i=1}^{N}{∥e_{i}\left(t\right)∥}_{1}^{\gamma }\leq -\beta {\left(\sum_{i=1}^{N}{∥e_{i}\left(t\right)∥}_{1}\right)}^{\gamma }. \end{array}$$

On account of Lemma 2, it has

(25) $$\begin{array}{c} -\frac{1}{2}\sum_{i=1}^{N}d_{i}\left({\left[e_{i}\left(t\right)\right]}^{H}e_{i}\left(t\right)+e_{i}^{H}\left(t\right)\left[e_{i}\left(t\right)\right]\right)=-\sum_{k=1}^{n}I_{N}^{T}D\underline{e_{k}\left(t\right)}, \end{array}$$

and

(26) $$\begin{array}{cc} & -\frac{1}{2}\sum_{r=1}^{2}\sum_{i=1}^{N}\varepsilon_{i}^{\left(r\right)}\left({\left[e_{i}\left(t-\tau_{r}\right)\right]}^{H}e_{i}\left(t-\tau_{r}\right)+e_{i}^{H}\left(t-\tau_{r}\right)\left[e_{i}\left(t-\tau_{r}\right)\right]\right)=-\sum_{r=1}^{2}\sum_{k=1}^{n}I_{N}^{T}\Pi^{\left(r\right)}\underline{e_{k}\left(t-\tau_{r}\right)}. \end{array}$$

Substituting (21)–(26) into (20), we derive

(27) $$\begin{array}{cc} {}_{t_{0}}^{C}D_{t}^{\alpha }V_{2}\left(t\right)\leq & \sum_{k=1}^{n}I_{N}^{T}\left(\eta_{1}E_{N}+\Xi^{\left(k\right)}-D\right)\underline{e_{k}\left(t\right)}+\sum_{k=1}^{n}I_{N}^{T}\left(\zeta_{1}E_{N}-\Pi^{\left(1\right)}\right)\underline{e_{k}\left(t-\tau_{1}\right)}\\ & +\sum_{k=1}^{n}I_{N}^{T}\left(\Omega^{\left(k\right)}-\Pi^{\left(2\right)}\right)\underline{e_{k}\left(t-\tau_{2}\right)}-\beta {\left(\sum_{i=1}^{N}{∥e_{i}\left(t\right)∥}_{1}\right)}^{\gamma }\\ \leq & -\beta {\left(\sum_{i=1}^{N}{∥e_{i}\left(t\right)∥}_{1}\right)}^{\gamma }. \end{array}$$

From Lemma 5, the FFCDN (3) under the controller (16) could achieve FNTS within time $T_{2}$. Theorem 2’s proof is now finished. □

**Remark** **5.**  *The positive constants $d_{i},\beta ,\varepsilon_{i}^{\left(1\right)},\varepsilon_{i}^{\left(2\right)},i=1,2,\cdots ,N$ in Theorems 1 and 2 can be flexibly selected within the range of satisfying the conditions (7) and (17). When we selected parameters within the required range, we tried to ensure that the system can show a chaotic state, so as to increase the complexity and reliability of the numerical simulation and make the complex dynamical networks closer to reality.*

**Remark** **6.**  *In the article, the FNTS issue for a class of FFCDNs with linearly non-delayed and delayed couplings is deliberated based on the quadratic norm and the absolute norm. From the limitation conditions 0<γ<2α−1 and 0<α<1 in Theorem 1, we have $\frac{1}{2}<\alpha <1$. However, in Theorem 2, only 0<γ<α and 0<α<1 are required. The FNTS conditions in Theorem 2 are less conservative. The STs $T_{1}$ and $T_{2}$ are related to the initial value of the system. In the process in practical applications, when the system is given a specific initial value, both controllers can be used. Select the controller under which the ST is shorter, so as to obtain a more accurate ST estimation. Therefore, under different conditions, we can select different controllers according to the actual need to achieve better effective FNTS.*

**Remark** **7.**  *It can been seen from inequalities (8) and (18) that the fractional-order of the system α and fractional-order power law β affect the upper bound of synchronous convergence time. In particular, $T_{1}$ and $T_{2}$ will monotonically increase with the increase of α or the decrease of β, which means that the FNTS effect is better when the fractional-order is small or the fractional-order power law is large.*

**Remark** **8.**  *The advantages of our primary results on FNTS of FFCDNs may be stated in three aspects when compared with existing approaches:*

*(1) There have been studies on fractional-order CV complex networks in recent years. However, in [33,34], the coupling strengths, inner coupling matrices, and outer coupling matrices were not considered as RV. In addition, for delayed fractional-order complex networks being fully CV in [36], internal delay and non-delayed and delayed couplings were not integrated into the model. Note that in this paper, the state variables, system function, coupling strengths, inner coupling matrices, and outer coupling matrices of directed complex dynamical networks (1) are all set as CV, which are different from the undirected complex networks without considering the internal delay and coupling delay proposed in [33,36]. The model in this paper is more practical and has a certain research value.*

*(2) In addition to the quadratic norm, the controller (6) is designed and the Lyapunov function is constructed based on the novel norm composed of the absolute values of the real and imaginary parts of the complex number. The FNTS criteria are obtained, and the ST is estimated. In contrast, the synchronization conditions obtained by the novel norm have a more comprehensive range of applications.*

*(3) In [25,26,27,28,29,30,31,32], the authors decomposed the fractional-order CV neural networks into two RV systems and obtained the stability criteria of the fractional-order complex-valued neural networks by studying the RV systems. Instead of the traditional technique of separating the complex-valued network into two parts, the main results in this paper were acquired by designing two delay-dependent controllers based on different norms, which effectively avoids the complexity of theoretical analysis caused by traditional separation methods.*

*Therefore, our findings are found to be an improvement of the previously published findings.*

**Remark** **9.**  *In the proof of Theorems 1 and 2, we used the inequality technique in Lemmas 1–4. This means that the value on the left of the inequality in Theorems 1 and 2 may be amplified to a certain extent, making the result conservative. On the other hand, the obtained results are independent of both internal delay and coupling delay, which cannot reflect the influences of delays on the synchronization effects. They are generally more conservative than delay-dependent criteria. Therefore, our future research will focus on improving Lemma 5 to reduce the conservatism of the results and obtain a more accurate ST.*

**Remark** **10.**  *The FNTS problem for a class of FFCDNs with internal delay and non-delayed and delayed couplings is studied by designing two different controllers in this paper. State variables, the system function, coupling strengths, inner coupling matrices, and outer coupling matrices in the delayed FFCDNs were all set as CV, which represents a more general situation. It can be applied to the analysis of dynamical networks with delay characteristics, such as confidential communication, image encryption, engineering control, urban transportation, communication engineering, bioengineering, etc.*

## 4. Numerical Simulations

A class of FFCDNs with four nodes including internal delay, no delay and delay coupling was considered.

(28) $$\begin{array}{cc} {}_{t_{0}}^{C}D_{t}^{\alpha }x_{k}\left(t\right)= & f\left(x_{k}\left(t\right),x_{k}\left(t-\tau_{1}\right)\right)+c_{1}\sum_{j=1}^{N}a_{kj}G_{1}x_{j}\left(t\right)+c_{2}\sum_{j=1}^{N}b_{kj}G_{2}x_{j}\left(t-\tau_{2}\right)+u_{k}\left(t\right), \end{array}$$

where $x_{k}\left(t\right)={\left(x_{k1}\left(t\right),x_{k2}\left(t\right),x_{k3}\left(t\right)\right)}^{T}$, k=1,2,3,4, $c_{1}=c_{2}=0.1+0.1i$, $G_{1}=G_{2}=\left(0.01-0.01i\right)E_{3}$, $f\left(x\left(t\right),x\left(t-\tau_{1}\right)\right)=D_{1}x\left(t\right)+g_{11}\left(x\left(t\right)\right)+g_{12}\left(x\left(t-\tau_{1}\right)\right)$, $g_{11}\left(x\right)=(0,-x_{1}x_{3},$$\frac{\bar{x_{1}}x_{2}+x_{1}\bar{x_{2}}}{2}{)}^{T}$, $g_{12}\left(x\right)={\left(0,\left(0.1+0.1i\right)x_{2},0\right)}^{T}$, $\tau_{1}=1.3$, $\tau_{2}=1$, and $A=\left(\begin{array}{cccc} -2-2i & 1+i & 0 & 1+i\\ 0 & 1+i & -1-i & 0\\ 1+i & 0 & -1-i & 0\\ 1+i & 0 & 0 & -1-i \end{array}\right)$, $B=\left(\begin{array}{cccc} 1-i & 0 & 0 & -1+i\\ 0 & 2-2i & -1+i & -1+i\\ 0 & 0 & 1-i & -1+i\\ -1+i & 0 & 0 & 1-i \end{array}\right)$,

$D_{1}=\left(\begin{array}{ccc} -35 & 35 & 0\\ -7 & 28 & 0\\ 0 & 0 & -3 \end{array}\right)$.

The dynamical behavior of the isolated node can be described by

(29) $$\begin{array}{ccc} {}_{t_{0}}^{C}D_{t}^{\alpha }s\left(t\right) & = & f(s\left(t\right),s\left(t-\tau_{1}\right)), \end{array}$$

where $s\left(t\right)={\left(s_{1}\left(t\right),s_{2}\left(t\right),s_{3}\left(t\right)\right)}^{T}\in C^{3}$.

**Figure 1:**
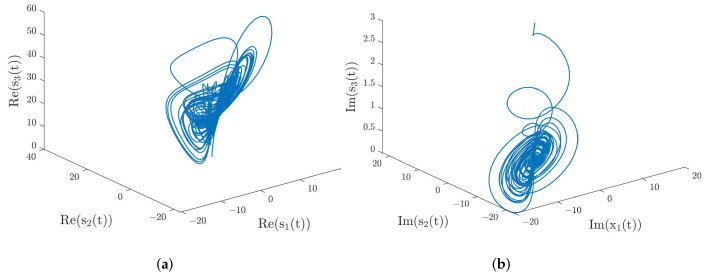
(**a**) Phase trajectories of the real parts of the system (29). (**b**) Phase trajectories of the imaginary parts of the system (29).

**Figure 2:**
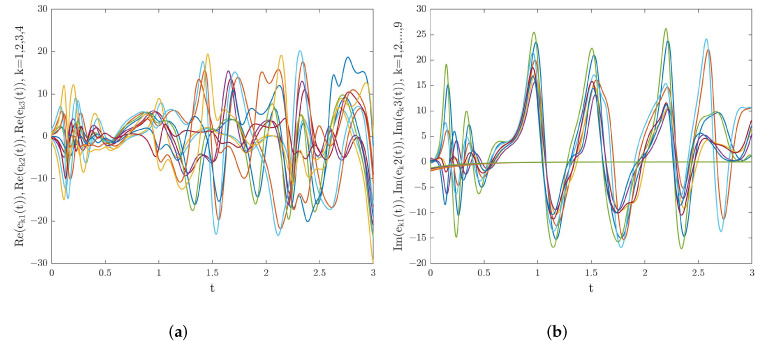
(**a**) Real part synchronization errors’ trajectories for system (28) without the controller. (**b**) Imaginary part synchronization errors’ trajectories for system (28) without the controller.

In the following numerical simulation, the initial conditions of the system (28) were selected as

(30) $$\left\{\begin{array}{c} x_{k1}\left(t\right)=1+0.3k+\left(1+0.4k\right)i,\\ \;x_{k2}\left(t\right)=1k+\left(2+0.1k\right)i,\\ x_{k3}\left(t\right)=2+0.5k+\left(1+0.2k\right)i, \end{array}\right.$$

where k=1,2,3,4, t∈[−1.3,0]. $s_{1}\left(t\right)=1.5+1.7i,s_{2}\left(t\right)=2.3+2.1i,s_{3}\left(t\right)=3+3i$, and t∈[−1.3,0] were taken as the initial conditions of the system (29). Figure 1 depicts the real and imaginary parts’ phase trajectories of the system (29) when α=0.98. Moreover, the trajectories of the synchronization errors of the system (28) are also shown in Figure 2 when there is no external control. From Figure 2, we can clearly see that system (28) cannot achieve synchronization when there is no control input.

**Figure 3:**
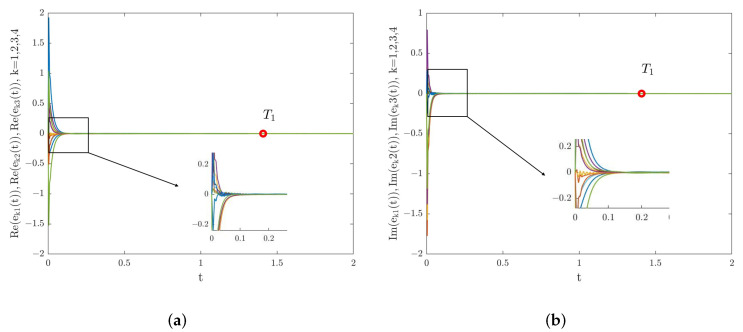
(**a**) Real part synchronization errors’ trajectories for system (28) under the controller (6). (**b**) Imaginary part synchronization errors’ trajectories for system (28) under the controller (6).

**Figure 4:**
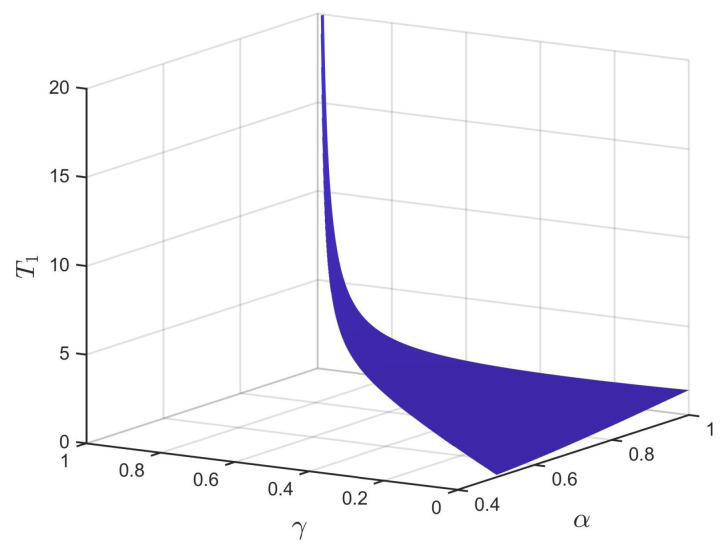
The relationship among the ST $T_{1}$, parameter α, and parameter γ.

**Figure 5:**
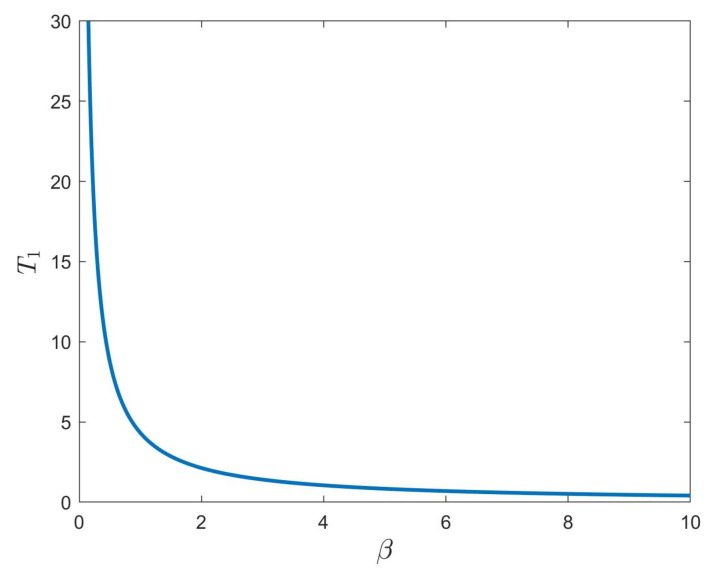
The relationship between the ST $T_{1}$ and parameter β.

First, consider the FNTS of the system (28) with the controller (6). Choose $d_{k}=55.8,\varepsilon_{k}^{\left(1\right)}=0.14,\varepsilon_{k}^{\left(2\right)}=0.005$, with k=1,2,3,4, α=0.98, γ=0.6, β=3. Then, the criteria in Theorem 1 are met. According to Theorem 1, system (28) can achieve FNTS under the controller (6), and the ST can be estimated as $T_{1}$ = 1.409. The trajectories of the synchronization errors for system (28) under the controller (6) are shown in Figure 3. Under the conditions of 0<γ<2α−1 and β=3, Figure 4 describes the relationship among the estimated ST $T_{1}$, the fractional order of the system α, and the control parameter ε. From Figure 4, we can see that $T_{1}$ increases with the increase of α. Besides, the estimated ST $T_{1}$ is also affected by the fractional-order power law β. Under the conditions of α=0.98 and γ=0.6, Figure 5 shows the relationship between the estimated ST $T_{1}$ and fractional-order power law β, that is $T_{1}$ will increase monotonically with the decrease of β.

**Figure 6:**
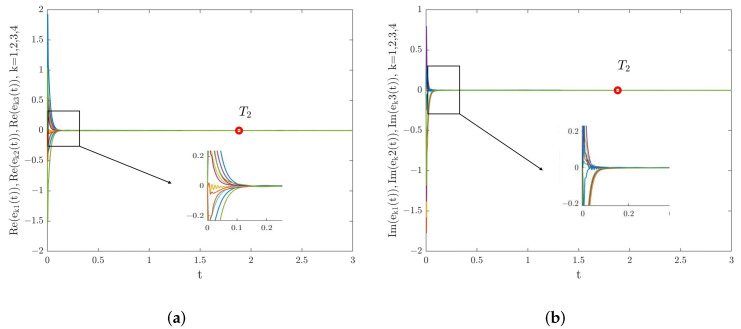
(**a**) Real part synchronization errors’ trajectories for system (28) under the controller (16). (**b**) Imaginary part synchronization errors’ trajectories for system (28) under the controller (16).

**Figure 7:**
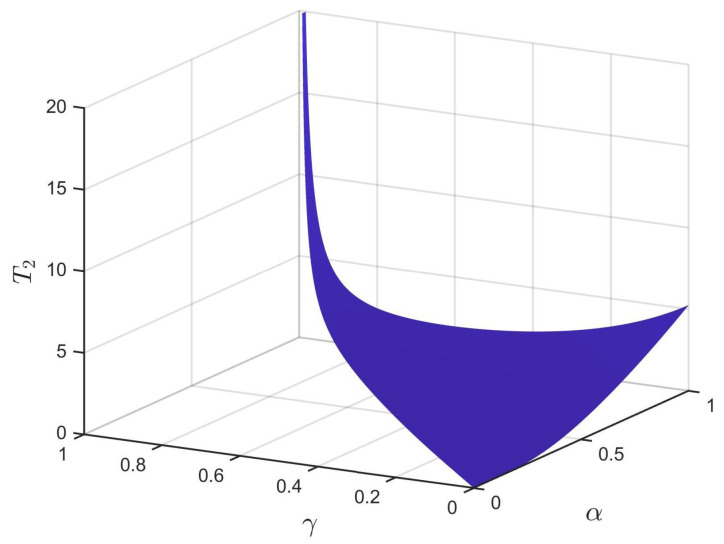
The relationship among the ST $T_{2}$, parameter α, and parameter γ.

**Figure 8:**
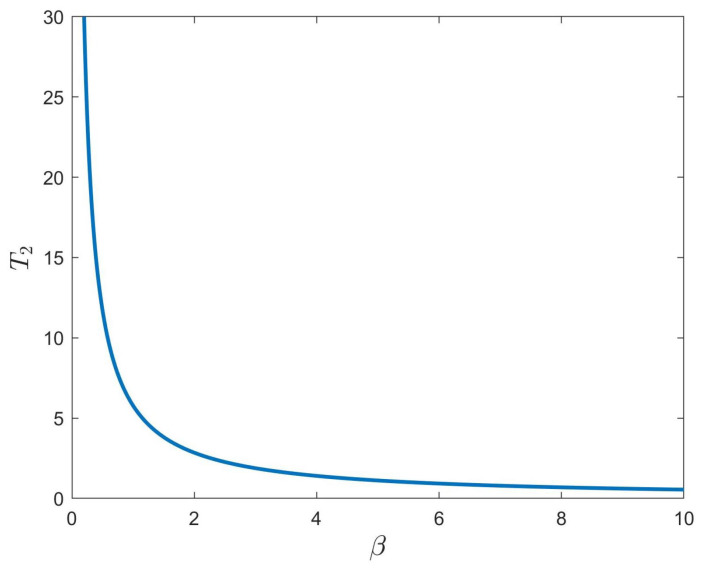
The relationship between the ST $T_{2}$ and parameter β.

Next, we analyze the FNTS of the system (28) with the controller (16). We chose $d_{k}=63.1,\varepsilon_{k}^{\left(1\right)}=0.2,\varepsilon_{k}^{\left(2\right)}=0.008$, with k=1,2,3,4. Then, condition (17) is satisfied. According to Theorem 2, system (28) is finite-time synchronized. Select α=0.98, γ=0.6, β=3; the trajectories for system (28) under the controller (16) are shown in Figure 6, where the ST can be estimated as $T_{2}$ = 1.8833. Under the conditions of 0<γ<α and β=3, Figure 7 describes the relationship between the estimated ST $T_{2}$, the fractional order of the system α, and the control parameter γ. From Figure 7, we can see that $T_{2}$ increases with the increase of α. Besides, the estimated ST $T_{2}$ is also affected by the fractional-order power law β. Under the conditions of α=0.98,γ=0.6, Figure 8 shows the relationship between the estimated ST $T_{2}$ and fractional-order power law β, that is $T_{2}$ will increase monotonically with the decrease of β.

In order to compare the performance of the controllers, the comparison of the settling times between the controllers (6) and (16) under the same parameters is shown in Table 2. From Table 2, we can see that under the initial value conditions given in this paper, the controller (6) performs better than the controller (16) with the same parameters, and the finite-time estimation is shorter. From Figure 4 and Figure 7 and Table 2, it can be seen that the ST for FNTS of the system (28) will increase with the increase in α or the decrease in β under both controllers. Therefore, for practical applications, the parameters can be selected according to different actual needs to obtain a better synchronization effect.

**Remark** **11.**  *To some extent, the estimated ST reflects the network’s synchronization rate. In this paper, the finite-time stability theory was used to estimate the ST of the system (28). It can be observed from Figure 3 and Figure 6 that the estimated ST is longer than the actual value. The estimated ST is conservative to some extent, indicating that there is an estimation error. The reason for the existence of the estimation error is that when we studied the FNTS criteria of the system (28), we used the inequality methods such as Lemmas 1–5 to amplify the result of ST estimation. Every scholar tries to find the minimum value of the ST, but such errors are inevitable. How to improve Lemma 5 and estimate the ST more accurately will be the focus of our future research.*

## 5. Conclusions

The FNTS problem for a class of FFCDNs with internal delay, as well as non-delayed and delayed couplings was studied in this research. In this paper, non-separation technology was used to study the FFCDNs. By designing two delay-dependent controllers based on different complex norms, some simple and useful synchronization criteria were obtained to guarantee the proposed dynamical networks could be FNTS. The current research highlights the importance of two parameters, the fractional order of system α and the fractional-order power law β, which would be the key parts in the prediction of the ST of FNTS. The research will help us better understand the impact of network structure and, consequently, find an effective way to improve the network performance. 

## Figures and Tables

**Table 1:** Comparisons with other similar papers.

Ref.	Fractional Order	Number Field	Non-Separation Method	Internal Delays	Coupling Delays	Types of Synchronization
[32]	✔	CV	✕	✕	✕	FNTS
[33]	✔	CV	✔	✕	✕	FNTS
[35]	✔	fully CV	✔	✕	✕	FNTS
[36]	✕	fully CV	✔	✕	✕	FNTS/FXTS
[41]	✕	fully CV	✔	✔	✕	FNTS
[42]	✕	fully CV	✔	✔	✕	FNTS/FXTS
[43]	✔	CV	✔	✔	✕	ADS
[44]	✕	CV	✔	✕	✕	FNTS
[45]	✕	RV	✕	✔	✕	FXTS
[46]	✕	RV	✕	✔	✔	FNTS
This paper	✔	fully CV	✔	✔	✔	FNTS

**Table 2:** Comparison of the settling times under different controllers.

	Controller (6)			Controller (16)		
α	0.98	0.98	0.87	0.98	0.98	0.87
β	2	3	3	2	3	3
γ	0.6	0.6	0.58	0.6	0.6	0.58
*T*	2.1311	1.409	0.9922	2.8484	1.8833	1.4002

## Data Availability

Not applicable.
